# The modified biochars influence nutrient and osmotic statuses and hormonal signaling of mint plants under fluoride and cadmium toxicities

**DOI:** 10.3389/fpls.2022.1064409

**Published:** 2022-12-12

**Authors:** Salar Farhangi-Abriz, Kazem Ghassemi-Golezani

**Affiliations:** Department of Plant Eco-physiology, Faculty of Agriculture, University of Tabriz, Tabriz, Iran

**Keywords:** chlorophyll, nutrients, plant growth, pollutants, proline, salicylic acid

## Abstract

**Introduction:**

Chemically modified biochars are a new generation of biochars that have a great ability to absorb and stabilize environmental pollutants. In this research, the physiological performance of mint plants (*Mentha crispa* L.) under fluoride and cadmium toxicities and biochar treatments was evaluated.

**Methods:**

Four levels of soil toxicities including non-toxic, 600 mg NaF kg^-1^ soil, 60 mg Cd kg^-1^ soil, and 600 mg NaF kg^-1^ soil + 60 mg Cd kg^-1^ soil were applied. The biochar addition to the soil was 25 g kg^-1^ (non-biochar, solid biochar, H_2_O_2_, KOH, and H_3_PO_4_-modified biochars).

**Results:**

The results showed that the application of biochar and especially chemically modified biochars reduced fluoride (about 15-37%) and cadmium (30-52%) contents in mint leaves, while increased soil pH and cation exchange capacity (CEC), nitrogen (12-35%), phosphorus (16-59%), potassium (17-52%), calcium (19-47%), magnesium (28-77%), iron (37-114%), zinc (45-226%), photosynthetic pigments of leaves and plant biomass (about 10-25%) under toxic conditions.

**Discussion:**

The biochar-related treatments reduced the osmotic stress and osmolytes content (proline, soluble proteins, and carbohydrates) in plant leaves. Plant leaf water content was increased by solid and modified biochar, up to 8% in toxic conditions. Furthermore, these treatments reduced the production of stress hormones [abscisic acid (27-55%), salicylic acid (31-50%), and jasmonic acid (6-12%)], but increased indole-3-acetic acid (14-31%) in plants under fluoride and cadmium stresses. Chemically modified biochars reduced fluoride and cadmium contents of plant leaves by about 20% and 22%, respectively, compared to solid biochar.

**Conclusion:**

This result clearly shows the superiority of modified biochars in protecting plants from soil pollutants.

## 1 Introduction

Soil contamination with environmental pollutants reduces plant growth and productivity and causes many risks to food security and human health in the world (Alengebawy et al., 2021). Fluoride and cadmium are important pollutants that are increasing in nature due to human activities such as mining and application of chemical fertilizers in agriculture (Choudhary et al., 2019; Yotsova et al., 2020). Typically, these two environmental pollutants are present in the soil at the same time and cause various physiological and biochemical disorders in plants (Li et al., 2018). The accumulation of fluoride and cadmium ions in the plant cells increases oxidative stress in plant tissues and causes damage to the cells (Sharma and Kaur, 2018; Shen et al., 2022). Another harmful effect of cadmium and fluoride in the soil is the reduction of water absorption by plants. These environmental pollutants cause a decrease in water absorption by plants due to damage to the root and its transmission channels. Ghassemi-Golezani and Farhangi-Abriz (2019) have reported that fluoride reduces water absorption by safflower plants *via* decreasing root growth. Cadmium toxicity reduces water uptake by plants (Haider et al., 2021a). This toxicity decreases stomata conductivity and the constant flow of water from the rhizosphere to the plant (El Rasafi et al., 2022). In addition to reducing water absorption, the toxicity of fluoride and cadmium in plant cells causes a reduction in the absorption of numerous nutrients needed for plant growth, such as potassium (K), calcium (Ca), and magnesium (mg) (Ghassemi-Golezani and Farhangi-Abriz, 2019; Zulfiqar et al., 2022). Accumulation of environmental pollutants in plant organs can cause hormonal changes in tissues. For example, the accumulation of cadmium in plant tissues causes an increase in the concentration of abscisic acid (ABA) and a decrease in indole acetic acid (IAA) in plants (Hu et al., 2020; Rolon-Cardenas et al., 2022). The increment of ABA content, which is a stress hormone, causes a decrease in the growth of plants. Researchers have proposed various methods such as biochar application as an effective method to reduce the impacts of cadmium and fluoride toxicity in plants (Ali et al., 2019; Ghassemi-Golezani and Farhangi-Abriz, 2019).

Biochar is a carbon-rich material obtained through pyrolysis of natural materials such as agricultural waste (Ghassemi-Golezani and Farhangi-Abriz, 2022; Waqar et al., 2022). This natural substance has special electrochemical properties that act as a soil amender. Having alkaline nature (in most cases), a high level of cation exchange capacity, pollutant adsorption capacity, and being rich in nutrients are among the important characteristics of this organic matter (Boateng et al., 2015; Batool et al., 2015; Javeed et al., 2022). Application of the biochar under different environmental stresses has improved the growth and physiological efficiency of plants. For example, adding biochar to the soil has improved plant growth under salt (Ghassemi-Golezani et al., 2021), copper (Rehman et al., 2019) cadmium (Mehmood et al., 2018; Haider et al., 2021b), fluoride (Ghassemi-Golezani and Farhangi-Abriz, 2019) and arsenic (Alam et al., 2019) stresses. Ghassemi-Golezani and Farhangi-Abriz (2019) reported that biochar addition to the soil reduces fluoride availability in the rhizosphere and increases potassium uptake by plants under fluoride toxicity of soil. Abbas et al. (2017) showed that application of biochar noticeably enhances wheat growth and productivity under cadmium toxicity. Rizwan et al. (2018) stated that biochar enhances the physiological performance of rice plants by improving water retention between rhizosphere and plants under cadmium stress. In a recent study, Ren et al. (2021) showed that biochar addition to the soil increases tobacco growth *via* decreasing cadmium accumulation in plant tissues and increasing photosynthetic activities under contaminated soil with cadmium. Biochar can improve the growth of plants under environmental pollution in two direct and indirect ways. The direct effect is related to increasing the adsorption and retention of pollutants on the surface of biochar and reducing their transmission to rhizosphere and plants (Park et al., 2011). While the indirect effect is related to the improvement of rhizosphere conditions, such as increasing soil pH, microbial activity, cation exchange capacity, and availability of nutrients (Medyńska-Juraszek et al., 2020; Farhangi-Abriz et al., 2022).

Researchers have proposed different methods to increase the performance of biochar in decreasing the harmful effects of environmental stresses on plants and improving the physicochemical conditions of the rhizosphere. Improving the performance of biochar with chemical reagents is one of the easiest and most practical methods (Liu et al., 2022). Typically, biochars engineered by chemical reagents have a higher cation exchange capacity, absorbance ability of environmental pollutants, and specific surface area (Tan et al., 2016; Ghassemi-Golezani and Farhangi-Abriz, 2022). For example, modification of biochar by iron particles and application of this modified biochar under arsenic toxicity enhanced rice growth more than solid biochar (Wen et al., 2021). Farhangi-Abriz and Ghassemi-Golezani (2021) reported that modified biochars with magnesium and manganese ions have an excellent sodium sorption capacity, which noticeably improves safflower growth and productivity under salt stress. In another study, Ghassemi-Golezani and Farhangi-Abriz (2022) stated that modification of biochar with chemical regents such as H_2_O_2_, KOH, and H_3_PO_4_ increases field capacity and available water content of rhizosphere for plants, more than solid biochar. In a recent study, Rahimzadeh and Ghassemi-Golezani (2022) showed that biochar modifications with zinc and iron particles improved dill performance under salt stress *via* increasing nutrient availability in rhizosphere and decreasing sodium uptake by plants. The majority of the advantageous effects of chemically modified biochars in pollutant adsorption are related to modifications in functional groups on the surface area of the biochar. According to Xue et al. (2012), modification of biochar with H_2_O_2_ boosted lead sorption from 0.88 to 22.82 mg g^-1^, compared to commercially activated carbon and increased oxygen-containing functional groups on the surface of the biochar, particularly carboxyl groups. Based on a report by Jin et al. (2014), arsenic adsorption by KOH-modified biochar in comparison with solid biochar increased by more than 1.3 times.

Mint (*Mentha crispa* L.) is a medicinal and industrial plant that can be cultivated under contaminated soils with pollutants for the production of essential oil. Mints are economically significant, because they are employed as flavoring agents in the food, and used as medicine, fragrance, insect repellant, detergents, and cosmetics all over the world (Brahmi et al., 2017). Typically, environmental pollutants such as heavy metals are inorganic compounds that cannot enter the essential oil. However, the presence of environmental pollutants can cause growth reduction and various physiological disorders in plants. Considering the effectiveness of chemically modified biochars in adsorbing environmental pollutants and increasing plant growth, this research has been carried out to investigate the possible effects of chemically modified biochars by H_2_O_2,_ KOH, and H_3_PO_4_ on the physiological efficiency of mint plants under fluoride and cadmium toxicities.

## 2 Materials and methods

### 2.1 Preparation of biochar and modified biochars

Solid biochar was obtained from a local company and then chemically modified by H_2_O_2_, KOH, and H_3_PO_4_, using standard methods. The method of Xue et al. (2012) was followed to prepare H_2_O_2-_modified biochar. In this method, 20 g of biochar were immersed in 200 ml of 30% hydrogen peroxide solution for 48 h at a temperature of 25°C. The method of Takaya et al. (2016) was applied to prepare KOH-modified biochar. About 20 g KOH were dissolved in 200 mL distilled water and 4 g biochar were added to the solution. The biochar was placed in the KOH solution for 24 hours at 25°C. To produce H_3_PO_4_-modified biochar, a 14% phosphoric acid solution was prepared. Subsequently, 200 g of biochar were added to 400 ml of H_3_PO_4_ solution. The biochar was kept in an H_3_PO_4_ solution for 24 hours at a temperature of 25°C. After mixing biochar with the chemical reagents, the pH of the modified biochar was adjusted to about the neutral pH (7-7.5). The various properties of solid and chemically modified biochars were determined by standard methods (White, 2010; Mohan et al., 2014; Xu et al., 2014; Graber et al., 2017; Lateef et al., 2019; Ghassemi-Golezani and Farhangi-Abriz, 2021) and presented in **Table 1**.

**Table 1:** Some physicochemical properties of the experimental soil, solid biochar and chemically modified biochars.

Soil		Solid biochar		H_2_O_2_ Modified biochar		KOH Modified biochar		H_3_PO_4_ Modified biochar	
Texture	Silty loam	N (%)	0.52	N (%)	0.51	N (%)	0.48	N (%)	0.48
pH	6.2	C (%)	44.1	C (%)	38.23	C (%)	38.37	C (%)	40.31
EC (dSm^−1^)	1.32	H (%)	2.4	H (%)	3.51	H (%)	2.85	H (%)	2.96
OC (g kg^-1^)	11.1	O (%)	27.40	O (%)	30.20	O (%)	31.89	O (%)	33.40
Total N (%)	0.09	P (mg kg^-1^)	8250	P (mg kg^-1^)	8120	P (mg kg^-1^)	8212	P (mg kg^-1^)	8940
P (mg kg^-1^)	31.2	S (mg kg^-1^)	5790	S (mg kg^-1^)	4340	S (mg kg^-1^)	4360	S (mg kg^-1^)	5100
K (mg kg^-1^)	187.1	Na (mg kg^-1^)	2.30	Na (mg kg^-1^)	1.34	Na (mg kg^-1^)	1.12	Na (mg kg^-1^)	1.35
Mg (mg kg^-1^)	70.4	K (mg kg^-1^)	4070	K (mg kg^-1^)	3823	K (mg kg^-1^)	4150	K (mg kg^-1^)	3560
Mn (mg kg^-1^)	10.80	Ca (mg kg^-1^)	3750	Ca (mg kg^-1^)	3021	Ca (mg kg^-1^)	2980	Ca (mg kg^-1^)	3090
SF (mg kg^-1^)	8.30	Mg (mg kg^-1^)	166.4	Mg (mg kg^-1^)	102.7	Mg (mg kg^-1^)	110.4	Mg (mg kg^-1^)	105.3
Cd (mg kg^-1^)	0.13	Mn (mg kg^-1^)	37.30	Mn (mg kg^-1^)	35.8	Mn (mg kg^-1^)	32.2	Mn (mg kg^-1^)	22.40
CEC (cmol kg^-1^)	18.80	Cl (mg kg^-1^)	N. A	Cl (mg kg^-1^)	N. A	Cl (mg kg^-1^)	N. A	Cl (mg kg^-1^)	N. A
Bulk density (g cm^-3^)	1.43	SF (mg kg^-1^)	5.2	SF (mg kg^-1^)	4.3	SF (mg kg^-1^)	3.4	SF (mg kg^-1^)	3.7
Moisture (%)	1	Cd (mg kg^-1^)	0.06	Cd (mg kg^-1^)	0.06	Cd (mg kg^-1^)	0.06	Cd (mg kg^-1^)	0.06
		CEC (cmol kg^-1^)	20.21	CEC (cmol kg^-1^)	24.60	CEC (cmol kg^-1^)	25.20	CEC (cmol kg^-1^)	26.10
		pH	7.1	pH	7.5	pH	7.5	pH	7.5
		S _BET_ (m^2^ g^-1^)	14.2	S _BET_ (m^2^ g^-1^)	48.1	S _BET_ (m^2^ g^-1^)	69.3	S _BET_ (m^2^ g^-1^)	77.3
		V_tot_ (cm^3^ g^-1^)	0.024	V_tot_ (cm^3^ g^-1^)	0.082	V_tot_ (cm^3^ g^-1^)	0.063	V_tot_ (cm^3^ g^-1^)	0.089
		AVP (nm)	3.43	AVP (nm)	3.57	AVP (nm)	3.22	AVP (nm)	3.24
		CA (mmol g^-1^)	0.21	CA (mmol g^-1^)	0.39	CA (mmol g^-1^)	0.45	CA (mmol g^-1^)	0.62
		LA (mmol g^-1^)	0.11	LA (mmol g^-1^)	0.19	LA (mmol g^-1^)	0.26	LA (mmol g^-1^)	0.18
		PA (mmol g^-1^)	0.68	PA (mmol g^-1^)	0.95	PA (mmol g^-1^)	1.32	PA (mmol g^-1^)	1.14
		F_ab_ (mg g^-1^)	3.41	F_ab_ (mg g^-1^)	4.59	F_ab_ (mg g^-1^)	4.43	F_ab_ (mg g^-1^)	5.17
		Cd _ab_ (mg g^-1^)	16.76	Cd _ab_ (mg g^-1^)	24.78	Cd _ab_ (mg g^-1^)	29.54	Cd _ab_ (mg g^-1^)	41.3
		Stability (◦C)	520	Stability (◦C)	505	Stability (◦C)	510	Stability (◦C)	505
		Moisture (%)	< 1	Moisture (%)	< 1	Moisture (%)	< 1	Moisture (%)	< 1

EC, Electrical conductivity; OC, Organic carbon; CEC, Cation exchange capacity; SF, Soluble fluoride; NA, Not available; F_ab_, Fluoride absorbing capacity; Cd _ab_, Cadmium absorbing capacity; S_BET_, BET surface area; V _tot_, Total pore volume; AVP, Average pore size, CA, Carboxylic acid groups; LA, Lactonic acid groups; PA, Phenolic acid groups.

### 2.2 Experimental conditions

Three experimental soil samples (specifications are presented in **Table 1**) were contaminated with pollutants (600 mg NaF kg^-1^ soil, 60 mg Cd kg^-1^ soil, and 600 mg NaF kg^-1^ soil + 60 mg Cd kg^-1^ soil) and another soil sample was not contaminated (non-toxic). Then, non-biochar and biochar-related treatments (solid biochar, modified biochars with H_2_O_2,_ KOH, and H_3_PO_4_) were applied. Contamination of soil with fluoride and cadmium ions was performed according Ghassemi-Golezani and Farhangi-Abriz (2019) and Cieśliński et al. (1996), respectively. Application rate of biochar was 25 g biochar per kg soil. About 3 kg of contaminated or non-contaminated soil was added to polyethylene pots (20 × 20 cm), using 60 pots in general. Thereafter, mint seeds were sown in each pot and irrigated with tap water up to 100% field capacity (FC) of growth media. The pots were kept in controlled greenhouse conditions for 45 days (day and night temperatures: 25 and 22°C, respectively; light intensity: 140 W m^-2^; and photoperiod: about 13 h.). Total soluble fluoride and cadmium concentrations in each pot were determined by the methods of Larsen and Widdowson (1971) and Lindsay and Norvell (1978), respectively. The pH of the soil was measured using a pH meter (Model: HI 99121, Hanna Instrument, USA), and the cation exchange capacity of the soil was quantified using the ammonium acetate method (Chapman, 1965). All growth and physiological measurements were performed 45 days after sowing.

### 2.3 Fluoride and cadmium content in mint leaves

The leaves were dried in an oven at 80°C for 48 hours and then powdered. The powdered leaves were digested in 0.1 N HCl at 25°C for 24 hours and were kept at 120°C for an hour. The digested materials were centrifuged at 25°C for 15 minutes at 5000 rpm. The supernatant was combined with distilled water and TISAB buffer. Then, fluoride content was measured, using the ion selectivity method (Orion 9609, Thermo Scientific, USA). The NaF with a 99.99% purity level served as the reference material for calculating fluoride levels in plant tissue (Sigma-Aldrich, United States). The atomic absorption spectrophotometry technique was used to determine the amount of cadmium accumulation in plant tissues.

### 2.4 Photosynthetic pigments

Chlorophylls and carotenoid contents were measured by Arnon (1949) and Maclachlan and Zalik (1963) methods, respectively. After homogenizing each sample (approximately 1 g) in 4 mL of acetone (80%), the samples were centrifuged at 12,000 g for 20 min at 4°C. A sample of the supernatant was obtained, and a spectrophotometer (Dynamica, Halo DB-20-UV-Visible Spectrophotometer, United Kingdom) was used to measure the absorbance at 645 and 663 nm (for chlorophylls) and 480 and 510 nm (for carotenoids). The total flavonoid in plant tissues was calculated following the Zhishen et al. (1999) method.

### 2.5 Nutrients content in mint leaves

The Kjeldahl method was used to assess the nitrogen level (Jones, 1991), and the yellow method and spectrophotometric analysis at 430 nm were used to determine the phosphorus concentration in mint leaves (Tandon et al., 1968). The contents of potassium, calcium, magnesium, iron, and zinc were determined in dried leaves. After being dry-ashed at 550°C for 7 hours, the plant leaves were digested in 5 M HNO_3_ and double-distilled water was added to achieve 50 mL. Then atomic absorption spectrophotometry was used to determine the cations (mg g^-1^ DW).

### 2.6 Leaf water content and osmolytes production

A plant was taken out of each pot, its leaves were separated, and they were weighed (FW). The leaves were then reweighed after being dried at 80°C for 48 hours (DW). The leaf water content (LWC) was calculated using the formula shown below:


LWC=[(FW-DW)/DW]×100

The proline content of mint leaves was determined according to the method described by Bates et al. (1973). First, 5 ml of 3% sulfosalicylic acid was used to homogenize 500 mg of fresh leaves. A plastic tube containing 2 ml of the extracted material was then filled with this mixture, along with 2 ml of glacial acetic acid and 2 ml of ninhydrin. The prepared samples were heated in a Bain Marie (BM-15 Bain Marie, Magapor SL, Spain) for an hour at 100°C. The mixture was then extracted with toluene once the sample was cooled at room temperature, and then the upper phase absorbance was read at 520 nm. By using the calibration curve for pure proline, the proline content of leaves was determined and expressed as mg g^-1^ fresh weight. The phenol-sulfuric acid and Bradford methods were applied to determine the contents of soluble sugars and proteins in mint leaves, respectively (Bradford, 1976; Kochert et al., 1978).

### 2.7 Hormones content in mint leaves

The ELISA method was applied to ascertain the endogenous concentrations of phytohormones including abscisic acid (ABA), indoleacetic acid (IAA), salicylic acid (SA), and jasmonic acid (JA). Initially, plant leaves were powdered and kept in the dark at 4°C for 24 hours. The powdered fresh samples (1 g each) were extracted in 80% cold methanol with butylated hydroxytoluene (BHT) as an antioxidant. Following the instructions provided in testing package, ELISA measurement was performed to assess the hormone content of leaves (Li and Meng, 1996; Wang et al., 2002).

### 2.8 Plant growth parameters

The plants of each pot were harvested separately and dried at 85°C for 48 hours to determine plant biomass. A portable leaf area meter (model ADCAM 300-United Kingdom) was used to measure the leaf area (LA) of the plants in each pot, and the results were reported as cm^2^ plant^-1^.

### 2.9 Statistical analysis

Using the MSTAT-C software from the East Lansing campus of Michigan State University in the United States, the data were analyzed on the basis of the experimental design (two-way ANOVA - factorial design based on randomized complete block arrangement with three repetitions). The Tukey HSD test was used to compare the means at p ≤ 0.05. Due to the large difference in the amount of cadmium in the soil and mint leaves under normal and stressful conditions, the statistical analysis of these traits has been done separately in each of the stressful and normal conditions. Figures were drawn by Excel 2019 from Microsoft Corporation, USA.

## 3 Results

### 3.1 Chemically modified biochar specifications

Leaching during the creation of modified biochars reduced the amounts of some nutrients such as calcium and magnesium in the biochar structure, whereas treatments with H_3_PO_4_ and KOH increased the contents of phosphorous and potassium in the biochar matrix (**Table 1**). In addition, modified biochars had higher oxygen and hydrogen percentages than solid biochar. Chemical treatments slightly decreased the stability of biochar. Modified biochars in comparison to solid biochar had higher CEC, Brunauer-Emmett-Teller (BET) surface area, total pore volume, average pore size, functional groups on biochar surface area, and fluoride and cadmium absorption capabilities. Modification of biochar by H_3_PO_4_, H_2_O_2_, and KOH increased the levels of carboxyl groups on biochar structure by about 195%, 114%, and 85%, respectively, compared to solid biochar. The KOH-treated biochar also had the greatest lactonic acids (about 136% more than solid biochar) and phenolic groups. The H_3_PO_4_-modified biochar had the highest amount of Brunauer-Emmett-Teller (BET) surface area, sorption capacity, and CEC, compared to solid biochar and other forms of chemically modified biochars.

### 3.2 Soil parameters

**Figure 1:**
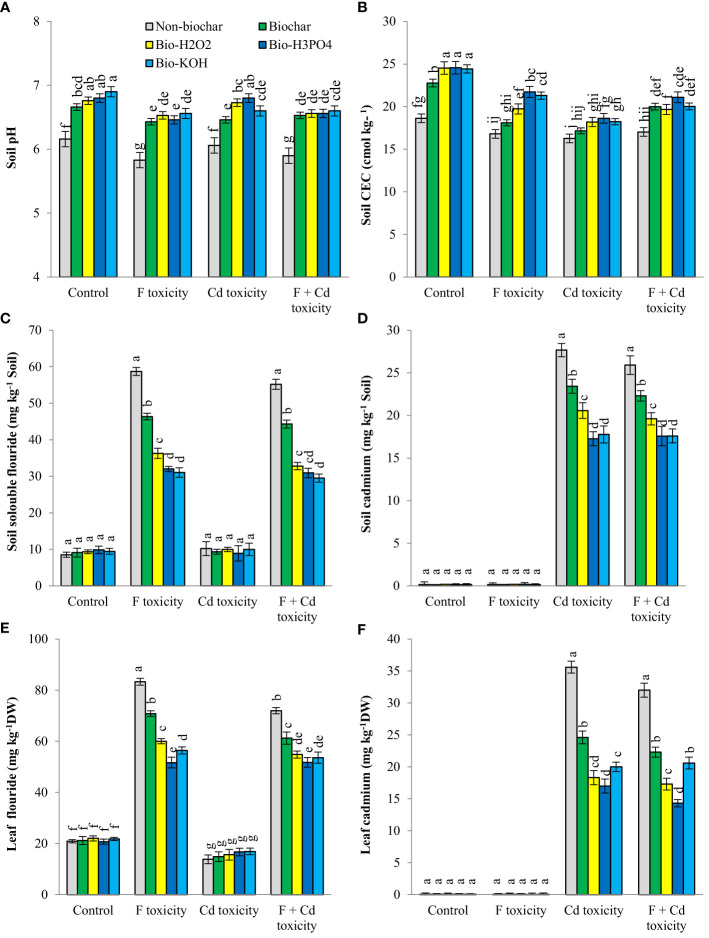
Changes in pH **(A)**, CEC **(B)**, fluoride **(C)** and cadmium **(D)** concentration of soil and fluoride **(E)** and cadmium **(F)** contents of leaves in response to biochar and chemically modified biochars under fluoride and cadmium toxicities. Data represent the average of three replicates (n = 3) ± standard error. Different letters indicate significant differences by Tukey HSD test at *p ≤ 0.05*. CEC: Cation exchange capacity; DW: dry weight; F: Fluoride; Cd: Cadmium; Bio-H_2_O_2_: Modified biochar by H_2_O_2_; Bio-KOH: Modified biochar by KOH; Bio-H_3_PO_4_: Modified biochar by H_3_PO_4_.

Contamination of the soil with fluoride and cadmium decreased pH (about 5% and 3%, respectively) and CEC (about 8% and 12%, respectively), whereas application of biochar and chemically modified biochars in rhizosphere increased these parameters (**Figure 1**). Under fluoride and fluoride + cadmium toxicities, enhancing soil pH by chemically modified biochars in comparison with solid biochar was statistically similar. However, chemically modified biochars had a better effect on increasing soil pH under cadmium toxicity, compared to solid biochar (about 5% more than solid biochar). Chemically modified biochars showed a similar effect on rising soil CEC under non-toxic conditions, but in toxic conditions, the H_3_PO_4_-modified biochar largely enhanced the soil CEC. For example, the H_3_PO_4_-modified biochar increased soil CEC under the combined form of cadmium and fluoride toxicities by about 23%, compared to the non-biochar treatment. Improvements of soil CEC by H_3_PO_4_-modified biochar under individual forms of cadmium and fluoride were about 12% and 21%, respectively.

Fluoride and cadmium availability in rhizosphere was significantly decreased by the addition of solid and modified biochars under both individual and combined forms of toxicities The availability of fluoride and cadmium under non-toxic conditions was unaffected by biochar treatments. In response to H_3_PO_4_ and KOH-modified biochars, the availability of fluoride under fluoride toxicity of soil decreased by around 45% and 47%, respectively, compared to non-biochar treatment. The H_3_PO_4_ and KOH-modified biochars in comparison with non-biochar treatment reduced cadmium availability by about 37% and 35%, respectively, under cadmium toxicity. Under the combined form of toxicities, the reduction of cadmium availability in soil caused by H_3_PO_4_ and KOH-modified biochars was about 32%.

### 3.3 Fluoride and cadmium contents in plant leaves

The content of fluoride and cadmium in mint leaves increased under individual and combined toxicity of these environmental pollutants (**Figure 1**). Under toxic conditions, the addition of solid and modified biochars to soil decreased the contents of fluoride and cadmium in mint leaves. However, biochar-related treatments did not change the fluoride and cadmium contents of leaves under non-toxic conditions. Chemically modified biochars were more successful than solid biochar in reducing fluoride (about 37%) and cadmium (about 27%) accumulation in plant leaves. Among the chemically modified biochars, The H_3_PO_4_ modified biochar was more successful in reducing the fluoride and cadmium in leaves. The reduction of fluoride in mint leaves by H_3_PO_4-_modified biochar under the individual and combined form of toxicities was about 38% and 28%, respectively. The decrement of cadmium toxicity by H_3_PO_4-_modified biochar under individual and combined forms of toxicities was about 41% and 55%, respectively.

### 3.4 Photosynthetic pigments

Fluoride and cadmium toxicities decreased the carotenoids (about 46% and 55%), flavonoids (about 57% and 64%), and chlorophylls contents (about 25% and 34%, respectively) in mint leaves (**Table 2**). The addition of solid and modified biochars significantly increased the photosynthetic pigments in mint plants under an individual and combined form of toxicities. However, biochar-related treatments did not have a tangible effect on photosynthetic pigments in non-toxic conditions. No significant difference was observed between different biochar treatments in increasing the content of photosynthetic pigments of mint leaves. The increment of total chlorophyll content in mint leaves by biochar-related treatments was about 17%, 27%, and 15%, under fluoride, cadmium, and fluoride + cadmium toxicities, respectively, compared to non-biochar treatment. Improvement of flavonoid and carotenoid contents of leaves by biochar treatments under combined form of cadmium and fluoride toxicities were about 58% and 32%, respectively.

**Table 2:** Means of plant biomass, leaf area and photosynthetic pigments in mint plants affected by biochar and chemically modified biochars under fluoride and cadmium toxicities.

Toxicity	Soil treatments	Plant biomass	Leaf area	Chl a	Chl b	Chl Total	Carotenoid	Flavonoid
		g plant^-1^	cm^2^ plant^-1^	mg g^-1^DW				
Non-toxic	Non-biochar	2.8 ± 0.09b	314.7 ± 2.90c	1.91 ± 0.08 a	0.93 ± 0.03 a	2.84 ± 0.07 a	0.63 ± 0.06 a	0.57 ± 0.05 ab
	Biochar	3.2 ± 0.08a	352.0 ± 4.27b	1.89 ± 0.05 a	0.96 ± 0.08 a	2.86 ± 0.11 a	0.58 ± 0.08 a	0.52 ± 0.03 b
	Biochar-H_2_O_2_	3.2 ± 0.06a	372.3 ± 3.68a	1.96 ± 0.09 a	0.95 ± 0.09 a	2.92 ± 0.08 a	0.60 ± 0.06 a	0.59 ± 0.04 a
	Biochar- H_3_PO_4_	3.3 ± 0.05a	383.7 ± 6.39a	1.97 ± 0.10 a	0.97 ± 0.04 a	2.95 ± 0.06 a	0.61 ± 0.05 a	0.53 ± 0.08 ab
	Biochar-KOH	3.2 ± 0.09a	384.0 ± 3.72a	2.01 ± 0.06 a	0.96 ± 0.02 a	2.94 ± 0.08 a	0.61 ± 0.04 a	0.54 ± 0.09 ab
Fluoride	Non-biochar	1.9 ± 0.06def	210.7 ± 5.81i	1.49 ± 0.05 e	0.62 ± 0.02 fg	2.11 ± 0.08 g	0.34 ± 0.07 d	0.24 ± 0.06 e
	Biochar	2.1 ± 0.09cd	241.7 ± 6.25efg	1.70 ± 0.11 bc	0.76 ± 0.08 bcd	2.46 ± 0.05 bcd	0.45 ± 0.09 bc	0.32 ± 0.04 cd
	Biochar-H_2_O_2_	2.1 ± 0.08cd	252.3 ± 7.31de	1.74 ± 0.06 bc	0.80 ± 0.08 b	2.53 ± 0.09 bc	0.46 ± 0.03 b	0.33 ± 0.03 cd
	Biochar- H_3_PO_4_	2.3 ± 0.11c	259.0 ± 3.93d	1.80 ± 0.09 b	0.78 ± 0.05 bc	2.57 ± 0.06 b	0.45 ± 0.09 bc	0.34 ± 0.05 cd
	Biochar-KOH	2.2 ± 0.08cd	251.0 ± 5.17def	1.76 ± 0.04 b	0.76 ± 0.09 bcd	2.52 ± 0.07 bc	0.44 ± 0.06 bc	0.33 ± 0.04 cd
Cadmium	Non-biochar	1.6 ± 0.04f	185.0 ± 2.88j	1.30 ± 0.06 f	0.55 ± 0.04 g	1.85 ± 0.05 h	0.28 ± 0.05 e	0.20 ± 0.04 e
	Biochar	1.8 ± 0.03def	222.3 ± 4.36hi	1.55 ± 0.09 de	0.72 ± 0.11 bcde	2.28 ± 0.05 ef	0.36 ± 0.06 d	0.32 ± 0.06 d
	Biochar-H_2_O_2_	1.9 ± 0.07cdef	241.3 ± 7.12efg	1.63 ± 0.05 cd	0.71 ± 0.05 cde	2.34 ± 0.09 de	0.36 ± 0.05 d	0.34 ± 0.09 cd
	Biochar- H_3_PO_4_	2.0 ± 0.09cde	245.0 ± 3.30def	1.66 ± 0.11 cd	0.72 ± 0.08 bcde	2.37 ± 0.12 cde	0.38 ± 0.08 d	0.33 ± 0.07 cd
	Biochar-KOH	1.9 ± 0.05cdef	245.0 ± 6.32def	1.65 ± 0.07 cd	0.69 ± 0.09 def	2.35 ± 0.07 de	0.40 ± 0.08 cd	0.34 ± 0.08 cd
Fluoride + Cadmium	Non-biochar	1.7 ± 0.07ef	192.0 ± 6.12j	1.48 ± 0.02 e	0.65 ± 0.04 ef	2.15 ± 0.06 fg	0.28 ± 0.04 e	0.24 ± 0.08 e
	Biochar	1.9 ± 0.07def	217.3 ± 4.32i	1.70 ± 0.08 bc	0.78 ± 0.02 bc	2.48 ± 0.05 bcd	0.35 ± 0.07 d	0.33 ± 0.09 cd
	Biochar-H_2_O_2_	2.1 ± 0.06cd	225.3 ± 7.39ghi	1.73 ± 0.04 bc	0.81 ± 0.05 b	2.52 ± 0.08 bc	0.37 ± 0.05 d	0.38 ± 0.08 c
	Biochar- H_3_PO_4_	1.8 ± 0.05def	234.3 ± 5.92fgh	1.74 ± 0.10 bc	0.80 ± 0.07 b	2.54 ± 0.09 bc	0.36 ± 0.07 d	0.34 ± 0.05 cd
	Biochar-KOH	2.1 ± 0.06cd	234.3 ± 4.38fgh	1.69 ± 0.07 bc	0.80 ± 0.08 b	2.52 ± 0.04 bc	0.36 ± 0.09 d	0.32 ± 0.09 cd

Data represents the average of three replicates (n=3) ± standard error. Different letters in each column indicate significant difference by Tukey HSD test at p ≤ 0.05. DW, Dry weight; Chl, Chlorophyll.

### 3.5 Nutrient content of plants

Fluoride and cadmium toxicities decreased the N (about 35% and 45%, respectively), P (about 58% and 53%), K (about 44% and 54%), Ca (about 49% and 54%), Mg (about 51% and 55%), Fe (about 57%) and Zn (about 57% and 74%) contents in mint leaves (**Table 3**). Soil treatment with solid and modified biochars noticeably improved the nutrient content of mint plants under normal and stressful conditions. However, the increment of N and P nutrients in response to biochar-related treatments under normal conditions was not statistically significant. Chemically modified biochars had better effects than solid biochar in increasing the leaf nutrients. In most cases, KOH and H_3_PO_4-_modified biochars had better effects than other biochar-related treatments in increasing the nutrients of mint leaves. Under the individual form of fluoride toxicity, the H_3_PO_4_-modified biochar increased N, P, K, Ca, Mg, Fe, and Zn contents in mint leave by about 22, 59, 35, 50, 56, 91 and 88%, compared to non-biochar treatment. The H_3_PO_4_-modified biochar under cadmium toxicity enhanced N, P, K, Ca, Mg, Fe, and Zn contents by about 35, 35, 52, 37, 77, 108, and 226%, respectively. Under the combined form of toxicities, increments in N, P, K, Ca, Mg, Fe, and Zn contents of mint leaves by H_3_PO_4_ modified biochar were about 21, 32, 25, 23, 38, 70, and 105%.

**Table 3:** Means of nutrients content in mint leaves affected by biochar and chemically modified biochars under fluoride and cadmium toxicities.

Toxicity	Soil treatments	Nitrogen	Phosphorous	Potassium	Calcium	Magnesium	Iron	Zinc
		mg g^-1^DW						
Non-toxic	Non-biochar	33.9 ± 0.78 a	21.9 ± 0.27 a	34.6 ± 1.26 c	11.2 ± 0.22 b	8.1 ± 0.19 c	0.83 ± 0.03 c	0.59 ± 0.02 b
	Biochar	35.2 ± 0.51 a	22.4 ± 0.52 a	39.9 ± 0.66 b	12.9 ± 0.66 a	9.1 ± 0.18 b	0.98 ± 0.09 b	0.68 ± 0.04 a
	Biochar-H_2_O_2_	34.1 ± 1.23 a	22.4 ± 0.34 a	42.8 ± 0.89 a	13.4 ± 0.65 a	9.9 ± 0.22 a	1.10 ± 0.08 a	0.69 ± 0.05 a
	Biochar- H_3_PO_4_	34.0 ± 0.89 a	22.0 ± 0.61 a	43.2 ± 0.92 a	13.6 ± 0.78 a	9.8 ± 0.22 ab	1.10 ± 0.08 a	0.71 ± 0.04 a
	Biochar-KOH	34.1 ± 0.66 a	22.1 ± 0.22 a	41.5 ± 0.83 ab	13.3 ± 0.92 a	9.9 ± 0.20 a	0.95 ± 0.05 b	0.68 ± 0.04 a
Fluoride	Non-biochar	21.9 ± 0.45 f	9.1 ± 0.21 i	19.2 ± 1.11 hi	5.7 ± 0.30 hi	3.9 ± 0.16 h	0.36 ± 0.06 h	0.25 ± 0.05 hi
	Biochar	24.7 ± 0.88 cde	12.2 ± 0.26 fg	23.6 ± 1.05 defg	7.0 ± 0.21 f	5.0 ± 0.22 g	0.54 ± 0.07 g	0.34 ± 0.03 fg
	Biochar-H_2_O_2_	26.4 ± 0.98 bc	14.3 ± 0.33 cd	25.1 ± 0.83 de	7.9 ± 0.16 de	5.8 ± 0.21 def	0.57 ± 0.08 efg	0.44 ± 0.03 cd
	Biochar- H_3_PO_4_	26.9 ± 0.46 b	14.5 ± 0.37 bcd	26.0 ± 0.62 d	8.6 ± 0.35 cd	6.1 ± 0.19 de	0.69 ± 0.03 de	0.47 ± 0.02 c
	Biochar-KOH	25.2 ± 0.76 bcd	14.4 ± 0.27 bcd	23.8 ± 0.51 efg	8.1 ± 0.38 cde	5.8 ± 0.18 def	0.68 ± 0.08 de	0.49 ± 0.07 c
Cadmium	Non-biochar	18.6 ± 0.92 g	10.3 ± 0.16 h	15.7 ± 0.88 j	5.1 ± 0.23 i	3.6 ± 0.33 h	0.35 ± 0.08 h	0.15 ± 0.03 j
	Biochar	23.0 ± 0.72 ef	12.0 ± 0.28 fg	20.8 ± 1.07 gh	6.1 ± 0.36 h	5.7 ± 0.24 efg	0.59 ± 0.05 efg	0.37 ± 0.04 ef
	Biochar-H_2_O_2_	24.0 ± 0.82 de	13.0 ± 0.43 ef	22.9 ± 1.21 efg	6.2 ± 0.41 ghi	6.2 ± 0.27 de	0.68 ± 0.03 de	0.48 ± 0.05 c
	Biochar- H_3_PO_4_	25.1 ± 0.79 bcd	13.9 ± 0.57 cde	23.9 ± 1.12 def	7.0 ± 0.25 f	6.4 ± 0.23 d	0.75 ± 0.04 cd	0.49 ± 0.06 c
	Biochar-KOH	25.2 ± 0.80 bcd	13.7 ± 0.60 de	23.7 ± 1.22 defg	6.9 ± 0.17 fg	6.3 ± 0.24 de	0.73 ± 0.05 cd	0.40 ± 0.07 de
Fluoride + Cadmium	Non-biochar	21.3 ± 0.69 f	11.3 ± 0.25 gh	18.1 ± 0.63 ij	6.1 ± 0.18 h	3.8 ± 0.24 h	0.40 ± 0.03 h	0.20 ± 0.03 ij
	Biochar	24.4 ± 0.73 de	13.8 ± 0.28 cde	21.2 ± 0.89 fgh	7.7 ± 0.23 e	5.1 ± 0.12 fg	0.55 ± 0.04 fg	0.29 ± 0.03 gh
	Biochar-H_2_O_2_	25.0 ± 0.39 bcd	14.8 ± 0.37 bcd	23.5 ± 0.82 defg	8.7 ± 0.26 c	5.6 ± 0.23 efg	0.58 ± 0.05 efg	0.38 ± 0.04 ef
	Biochar- H_3_PO_4_	25.8 ± 0.46 bcd	15.0 ± 0.28 bc	22.8 ± 0.72 efg	8.0 ± 0.28 cde	6.2 ± 0.24 de	0.68 ± 0.05 de	0.41 ± 0.05 de
	Biochar-KOH	25.9 ± 0.67 bcd	15.3 ± 0.48 b	23.6 ± 0.59 defg	8.0 ± 0.19 cde	6.3 ± 0.28 de	0.66 ± 0.04 def	0.40 ± 0.03 de

Data represents the average of three replicates (n=3) ± standard error. Different letters in each column indicate significant difference by Tukey HSD test at p ≤ 0.05. DW, Dry weight.

### 3.6 Leaf water content and osmolytes

**Figure 2:**
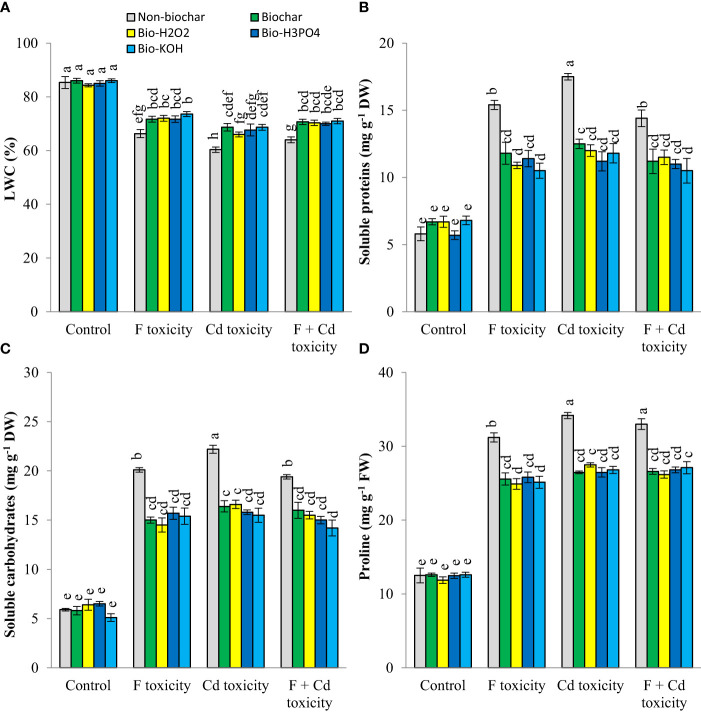
Changes in water content **(A)**, soluble proteins **(B)** carbohydrates **(C)** and proline **(D)** contents of mint leaves in response to biochar and chemically modified biochars under fluoride and cadmium toxicities. Data represent the average of three replicates (n = 3) ± standard error. Different letters indicate significant differences by Tukey HSD test at p ≤ 0.05. LWC: Leaf water content; F: Fluoride; Cd: Cadmium; Bio-H2O2: Modified biochar by H_2_O_2_; Bio-KOH: Modified biochar by KOH; Bio-H_3_PO_4_: Modified biochar by H_3_PO_4_.

Leaf water content was decreased by fluoride (by about 19%) and cadmium (by about 25%) toxicities (**Figure 2**). However, soluble proteins, carbohydrates, and proline production in mint plants were increased under toxic conditions. The addition of solid and modified biochars to the soil significantly increased the leaf water content of mint plants under toxic conditions, with no tangible effect in non-toxic condition. These improvements in leaf water content by biochar-related treatments were up to 7.3%, 8.3%, and 7% at fluoride, cadmium, and fluoride + cadmium toxicities, respectively. Under toxic conditions, biochar-related treatments decreased osmolyte production in mint leaves. These treatments similarly reduced the production of osmotic regulators. Decrement of soluble proteins, carbohydrates, and proline by biochar-related treatments under both cadmium and fluoride toxicities was up to 27%, 26%, and 19%, respectively, compared to non-biochar conditions.

### 3.7 Phytohormones

**Figure 3:**
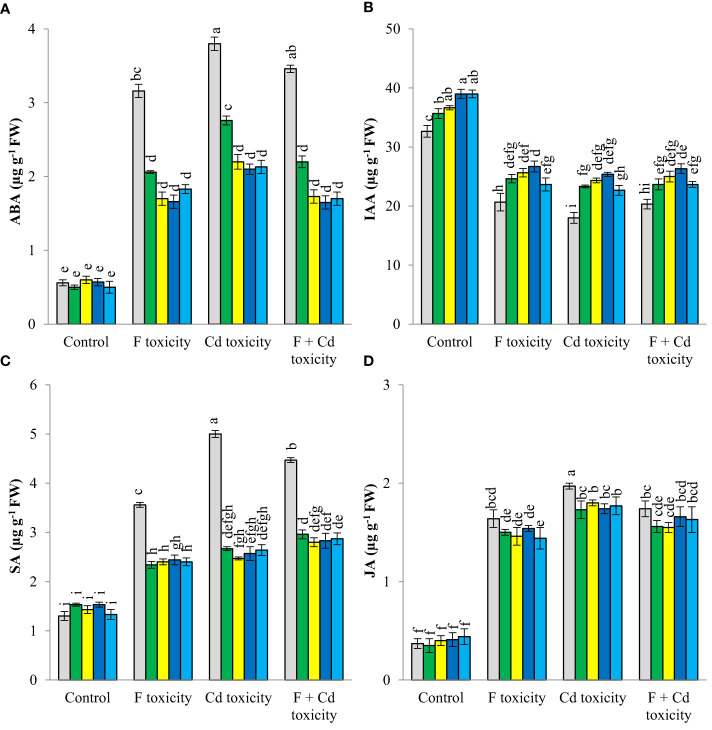
Changes in ABA **(A)**, IAA **(B)** SA **(C)** and JA **(D)** content of mint leaves in response to biochar and chemically modified biochars under fluoride and cadmium toxicities. Data represent the average of three replicates (n = 3) ± standard error. Different letters indicate significant differences by Tukey HSD test at p ≤ 0.05. ABA: abscisic acid content; IAA: indole-3-acetic acid; SA: salicylic acid; JA: jasmonic acid F: Fluoride; Cd: Cadmium; Bio-H_2_O_2_: Modified biochar by H_2_O_2_; Bio-KOH: Modified biochar by KOH; Bio-H_3_PO_4_: Modified biochar by H_3_PO_4_.

Fluoride and cadmium toxicities increased ABA (about 462% and 578%, respectively), SA (about 173% and 231%), and JA (about 343% and 432%) in mint leaves (**Figure 3**). However, IAA content was reduced by individual fluoride (about 36%) and cadmium (about 44%) toxicities. The reduction of IAA in mint leaves under the combined form of toxicities was about 37%, Under fluoride and cadmium toxicities, soil treatment with solid and chemically modified biochars increased IAA content, but decreased ABA, SA, and JA productions in mint leaves. Biochar-related treatments did not affect the ABA, SA, and JA contents of plants under non-toxic conditions, but increased IAA content in this condition. Chemically modified biochars were more successful than solid biochar in decreasing the ABA content of mint leaves under different toxic conditions. No significant difference was observed between chemically modified biochars in reducing the abscisic acid content of plants under stressful conditions. Decrement of ABA content of mint leaves by H_3_PO_4_ modified biochar was about 47%, 43%, and 50%, under fluoride, cadmium, and fluoride + cadmium toxicities, respectively. In most cases, H_3_PO_4_ and H_2_O_2_ modified biochars caused the highest increment in IAA production in mint leaves under all toxic and non-toxic conditions. The H_3_PO_4_-modified biochar increased the IAA content of mint leaves by about 19, 30, 41, and 29%, under non-toxic, fluoride, cadmium, and combined forms of toxicities, respectively. All biochar-related treatments had similar effects in reducing jasmonic and salicylic acids under different stressful conditions.

### 3.8 Plant growth

The mint biomass was lowered by cadmium (about 42%) and fluoride (about 32%) toxicities (**Table 2**). In both toxic and non-toxic conditions, solid and modified biochars increased plant biomass. Under toxic and non-toxic conditions, chemically modified biochars enhanced dry matter accumulation in mint plants, although in some cases this enhancement was not statistically significant, compared to solid biochar. Enhancement of dry mass production of mint plants by biochar-related treatments under fluoride, cadmium, and fluoride + cadmium toxicities was up to 21%, 25%, and 23%, respectively, compared to non-biochar treatment.

Mint leaf area was decreased by cadmium (by about 41%) and fluoride (by about 33%) toxicities, while soil treatment with biochar and modified biochars noticeably increased leaf expansion under both toxic and non-toxic conditions (**Table 2**). Chemically modified biochars were successful in developing the largest leaf area in plants. There was no statistically significant difference between the chemically modified biochars in terms of plant leaf area. Improvement of leaf area by the H_3_PO_4_ modified biochar was about 21%, 23%, 24%, and 17% under non-toxic, fluoride, cadmium, and fluoride + cadmium toxicities, respectively.

## 4 Discussion

The results of this research showed that the production and application of chemically modified biochars have a better effect than solid biochar in increasing the growth of plants under fluoride and cadmium toxicities. The biochar-related treatments improved the growth of plants by reducing fluoride and cadmium availabilities in the rhizosphere (**Figure 1**) and increasing the availability of water (**Figure 2**) and nutrients (**Table 3**) for plants. The pH and cation exchange capacity of the soil were raised by the biochar. The increment of soil pH by biochar is related to the alkaline nature of this organic matter. Typically, biochars have a more alkaline nature than soil, and their addition to soil increases soil pH. Improving the cation exchange capacity of the soil in response to biochar-related treatments is associated with increasing the injection of nutrients such as calcium and magnesium in the rhizosphere. Biochar is rich in nutrients, that improves soil fertility. The effect of biochar in improving the cation exchange capacity of soil can be related to an increase in soil pH. Previous studies have shown that there is a positive correlation between the increment of pH and the cation exchange capacity of the soil (Chintala et al., 2014; Ghassemi-Golezani and Farhangi-Abriz, 2019). Hailegnaw et al. (2019) stated that the addition of 8% biochar to the soil noticeably increases soil pH, CEC, and exchangeable calcium content. The increment in cation exchange capacity of soil treated with chemically modified biochars was higher than solid biochar, which could be associated with the higher specific surface area and cation exchange capacity of chemically modified biochars (**Table 1**).

The availability of environmental pollutants such as fluoride and cadmium in the rhizosphere is controlled by different environmental parameters such as the organic matter of soil and pH. The addition of biochar, especially chemically modified biochars, reduced the availability of cadmium and fluoride in the rhizosphere, leading to a reduction of these pollutants in the leaves of the plants (**Figure 1**). Biochar could reduce the absorption of environmental pollutants such as cadmium and fluoride by plants through two mechanisms. First, increasing soil pH reduces the availability of cadmium and fluoride and their absorption by plants (Ghassemi-Golezani and Farhangi-Abriz, 2019; Jing et al., 2020). Second, biochars, especially chemically modified biochars, have different functional groups on their surface area (Liu et al., 2022) (**Table 1**), which increase the surface adsorption of cadmium and fluoride by biochar and ultimately reduce the absorption of these elements by the plant. Zong et al. (2021) proved that number of functional groups on biochar surface area is one of the main factors in controlling cadmium adsorption by this organic material. The reason for the better performance of chemically modified biochars in reducing the availability of cadmium and fluoride in the soil (**Figure 1**) is related to their high adsorption capacity (**Table 1**), compared to solid biochar (**Table 1**). Modification of biochar by chemical reagents increases the number of functional groups on its surface area, which ultimately improves the adsorption capacity of this organic material (Li et al., 2020; Liu et al., 2022). In a recent study, Taqdees et al. (2022) showed that biochar modification with zinc particles improved radish growth under salt stress *via* decreasing sodium uptake by plants and enriching plant cells with zinc cations. In another study, Wen et al. (2021) reported that soil treatment by iron-modified biochar increases the cadmium sorption capacity of biochar, plant growth, and soil health in contaminated soils.

Destruction of photosynthetic pigments and reduction of their synthesis in plant leaves is one of the damaging effects of fluoride and cadmium stresses in plant leaves (Ghassemi-Golezani and Farhangi-Abriz, 2019; Chtouki et al., 2021). Cadmium and fluoride stresses in plant cells increase the activity of the chlorophyllase enzyme, which eventually destroys the chlorophyll structure (Sanjoy et al., 2011; Yadu et al., 2016). In addition, cadmium and fluoride toxicities increase reactive oxygen species, which have a high ability to destroy biological membranes. On the other hand, the decrease in chlorophyll synthesis in leaves under fluoride and cadmium stresses can be related to the reduction of some important nutrients such as iron and magnesium in leaves (**Table 3**). Magnesium is considered as a structural nutrient in the chlorophyll molecule, and iron is also a nutrient that plays a critical role in the synthesis of chlorophyll (Fageria, 2016). The decrease in the production of photosynthetic pigments under fluoride and cadmium toxicity has also been reported by other researchers (Lagriffoul et al., 1998; Ghassemi-Golezani and Farhangi-Abriz, 2019). Adding biochar to the soil improved the synthesis of photosynthetic pigments in mint leaves under fluoride and cadmium stress (**Table 2**). Solid and modified biochars reduced the harmful effects of these pollutants on the synthesis of chlorophyll by decreasing the absorption of fluoride and cadmium by plants. The biochar-related treatments may be also improved the synthesis of chlorophyll by increasing the absorption of various nutrients such as magnesium and iron. In addition, these treatments increased the nitrogen of leaves under fluoride and cadmium stress (**Table 3**), then can enhance chlorophyll synthesis. Researchers have reported a very strong correlation between leaf nitrogen content and chlorophyll synthesis (Ahanger et al., 2021). Another mechanism through which biochar can increase chlorophyll synthesis is the reduction of abscisic acid production under stress. Biochar treatments may also reduce chlorophyll destruction and helps its synthesis (**Figure 3**). An increase in the concentration of abscisic acid in the leaves engages the activation of enzymes such as chlorophyllase in the leaves, which ultimately causes a decrease in photosynthetic pigments of the leaves (Gupta et al., 2012). Zhu et al. (2020) reported that cadmium toxicity reduces chlorophyll content and photosynthetic activities in cotton leaves, but soil treatment with biochar mitigates the harmful effects of cadmium toxicity and increases chlorophyll biosynthesis in leaves.

Reduction of nutrient absorption by mint plants under fluoride and cadmium stresses is related to damage of root structure and cells. Cadmium and fluoride can reduce root growth and its absorption capacity (Dell Amico et al., 2008; Ghassemi-Golezani and Farhangi-Abriz, 2019; Shiyu et al., 2020). Cadmium can cause damage to the ion channels in the roots and ultimately reduce the absorption of nutrients by plants (Li et al., 2012). In addition, cadmium can compete directly with nutrients such as zinc in the rhizosphere and reduce their absorption by plants (Li et al., 2017). The increment of root cell lignification is another reason for reducing nutrient uptake by plants under cadmium toxicity. Lignification is a physiological process that blocks apoplastic pathways in plant roots and consequently decreases nutrient uptake by plants (Finger-Teixeira et al., 2010; Lux et al., 2011). Several reports have pointed out the reduction of the absorption of nutrients by plants under fluoride and cadmium toxicities (Ghassemi-Golezani and Farhangi-Abriz, 2019; Shiyu et al., 2020). Biochar is an organic soil amender that can act as an organic fertilizer in addition to the ability to regulate the physicochemical properties of the soil. This organic material with its high cation exchange capacity and specific surface area has a great ability to release nutrients into the rhizosphere. Typically, biochar has a high content of different macro and micronutrients, although the amount of these nutrients depends on the type of raw material from which biochar is produced. Increments of nutrients in plant tissues by biochar treatments under fluoride and cadmium toxicities (**Table 3**) can be related to the improvement of the physicochemical conditions of the rhizosphere and plant roots. Biochar and especially chemically modified biochars have a great cation exchange capacity (**Table 1**) that can improve the conditions of the rhizosphere for better absorption of nutrients by plants. In addition, these treatments can help to increase the water-holding capacity (Ghassemi-Golezani and Farhangi-Abriz, 2022) and microbial activities of the soil (Steinbeiss et al., 2009), which ultimately improves the bioavailability of the nutrients for plants.

Cadmium and fluoride stresses can cause less water absorption by the plant through the reduction of plant root growth (Ghassemi-Golezani and Farhangi-Abriz, 2019; Naeem et al., 2019). In addition, environmental stresses *via* increasing abscisic acid in the leaves and closing the leaf stomata can reduce the integrated movement of water from the rhizosphere to the plant and finally to the atmosphere, which imposes osmotic stress on plants (Kastori et al., 1992; Tao et al., 2021). Biochar is an organic material with high porosity that can increase the water-holding capacity of the soil and improve the available water for plants. The improvement of the water content of leaves under fluoride and cadmium toxicity in response to biochar can be related to an increase in plant root growth (Ghassemi-Golezani and Farhangi-Abriz, 2019). To deal with osmotic stress, plants increase the content of osmolytes in their tissues, so that they can adjust their osmotic pressure potential to continue water absorption (Farhangi-Abriz and Ghassemi-Golezani, 2018). The reduction of osmolytes in plants due to biochar-related treatments under fluoride and cadmium stresses (**Figure 2**) is related to a decrease in fluoride, cadmium, and endogenous stress hormones (ABA, SA, and JA) in the plant tissues. Stress hormones have a stimulating effect on the synthesis of osmolytes, which increases the content of these organic materials in plant tissues. Reduction of fluoride and cadmium absorptions by plants under biochar-related treatments was led to a decrease in osmolytes and stress levels.

Enhancing the concentration of stress hormones in plant tissues is a known physiological reaction to the environment, that can increase plant resistance to stress. Adding solid and modified biochars to the soil reduced the stress hormones (ABA, SA, and JA), and at the same time increased the growth-promoting hormone (IAA) in leaf tissues (**Figure 3**). The reduction of stress hormones due to biochar treatments can be related to the reduction of fluoride and cadmium entry into the plant tissues (**Figure 1**). An increase in the content of IAA in plant tissues by biochar treatments could be associated with a decrease in the amount of fluoride and cadmium accumulation in plant tissues. In addition, biochar treatments increased the concentration of zinc in leaf tissues (**Table 3**), which has a very critical role in the synthesis of auxins (Fageria, 2016).

Increasing cadmium and fluoride in plant tissues, osmotic stress, and stress hormones caused a decrease in the growth of plants under stress (**Table 2**). Adding solid and modified biochars to the soil improved plant growth under fluoride and cadmium stresses. Biochar-related treatments increased plant growth by reducing the harmful effects of fluoride and cadmium in plant tissues. These treatments improved the water content of leaves by decreasing abscisic acid in plant tissues. Having enough water is necessary for the optimal growth of the plant. Many physiological reactions of plants are carried out in the presence of water, which is essential for cell growth and division (Skirycz and Inzé, 2010). On the other hand, biochar treatments enhanced the absorption of many key nutrients for plant growth, such as nitrogen (a key nutrient in the structure of proteins), phosphorus (a nutrient that plays a role in plant energy storage), potassium (a nutrient that activates many enzymes), calcium (a nutrient effective in improving cell division), magnesium (an active nutrient in improving the synthesis of sugars and forming the structure of chlorophyll), iron (an active nutrient in oxidation and reduction reactions in plants and helping to build chlorophyll) and zinc (a nutrient that controls the production of auxins in the tissues) (Fageria, 2016), leading to better growth of plants under fluoride and cadmium toxicities. In addition, biochar increased the concentration of auxin (IAA) in the leaf tissue (**Figure 3**), which in turn improved plant growth and biomass.

## 5 Conclusion

Biochar and especially chemically modified biochars have a very good potential to reduce the absorption of fluoride and cadmium in plant tissues. These organic soil amenders improved the absorption of nutrients and water by plants under fluoride and cadmium stresses, causing a higher synthesis of photosynthetic pigments, leaf expansion, and growth. In addition, biochar-related treatments affected hormonal signaling in plants by reducing the content of growth retardant hormones and enhancing a growth-promoting hormone (IAA) in plant tissues. The results of this research confirm the superiority of chemically modified biochars in reducing the harmful effects of fluoride and cadmium toxicities on plants due to their specific physicochemical properties such as a high number of functional groups, specific surface area, and adsorption capacity. Therefore, chemically modified biochars can be used as a powerful soil amender in contaminated soils.

## Data availability statement

The original contributions presented in the study are included in the article/supplementary material. Further inquiries can be directed to the corresponding author.

## Author contributions

SF-A: Experimental work, Data analyzing, Writing. K-GG: Supervision, Experimental design, Writing. All authors contributed to the article and approved the submitted version.
